# Cardiorespiratory dynamics of type 2 diabetes mellitus: An extensive view of breathing and fitness challenges in a diabetes prevalent population

**DOI:** 10.1371/journal.pone.0303564

**Published:** 2024-07-05

**Authors:** Uzair Abbas, Shahbaz Ali Shah, Nisha Babar, Pashmina Agha, Mohiba Ali Khowaja, Maryam Nasrumminallah, Hibba Erum Arif, Niaz Hussain, Syed Mustafa Hasan, Israr Ahmed Baloch

**Affiliations:** 1 Dow University of Health Sciences, Karachi, Pakistan; 2 Worcestershire Acute Hospitals, NHS Trust, Worcestershire, United Kingdom; 3 Aga Khan University Hospital, Karachi, Pakistan; 4 Liaquat University of Medical and Health Sciences, Jamshoro, Pakistan; 5 Indus University, Karachi, Pakistan; 6 Lehigh Valley Hospital Cedar Crest, Allentown, Pennsylvania, United States of America; Hamasaki Clinic, JAPAN

## Abstract

**Background:**

Diabetes mellitus (DM) is well known for related micro and macrovascular complications. Uncontrolled hyperglycemia in diabetes mellitus leads to endothelial dysfunction, inflammation, microvascular impairment, myocardial dysfunction, and skeletal muscle changes which affect multiple organ systems. This study was designed to take an extensive view of cardiorespiratory dynamics in patients with type 2 DM.

**Methods:**

One hundred healthy controls (HC) and 100 DM patients were enrolled. We measured and compared the breathing patterns (spirometry), VO_2_ max levels (heart rate ratio method) and self-reported fitness level (international fitness scale) of individuals with and without diabetes. Data was analyzed in SPSS v.22 and GraphPad Prism v8.0.

**Results:**

We observed restrictive spirometry patterns (FVC <80%) in 22% of DM as compared to 2% in HC (p = 0.021). There was low mean VO_2_ max in DM as compared to HC(32.03 ± 5.36 vs 41.91 ± 7.98 ml/kg/min; p value <0.001). When evaluating physical fitness on self-reported IFiS scale, 90% of the HC report average, good, or very good fitness levels. In contrast, only 45% of the DM shared this pattern, with a 53% proportion perceiving their fitness as poor or very poor (p = <0.05). Restrictive respiratory pattern, low VO_2_ max and fitness level were significantly associated with HbA1c and long-standing DM.

**Conclusion:**

This study shows decreased pulmonary functions, decreased cardiorespiratory fitness (VO_2_ max) and IFiS scale variables in diabetic population as compared to healthy controls which are also associated with glycemic levels and long-standing DM. Screening for pulmonary functions can aid optimum management in this population.

## Background

Type 2 Diabetes Mellitus (T2DM) is characterized by insufficient insulin secretion by pancreatic β-cells and impaired responsiveness of insulin-sensitive tissues [1]. Globally, the prevalence of diabetes contributes to increased morbidity and mortality, making it a major public health concern [2]. According to the latest International Diabetes Federation (IDF), the global prevalence of type 2 diabetes mellitus (T2DM) in adults was 536.6 million people (10.5%) in 2021, and that there would be 783.2 million people (12.2%) living with diabetes worldwide by 2045 [3]. In Pakistan, 26.7% of adults in the year 2022 were affected by diabetes making the total number of approximately 33 million cases [4].

Diabetes is a micro-macrovascular disorder with debilitating effects on many organs including the development of nephropathy, retinopathy, neuropathy, along with cardiovascular abnormalities [5]. A number of studies have shown fibrotic changes in the lungs and pulmonary microcirculation disorders in patients with diabetes [6, 7]. Reduced elastic recoil, reduced lung volume, diminished respiratory muscle performance, chronic low grade inflammation, decrease in pulmonary diffusion capacity for carbon monoxide, autonomic neuropathy involving respiratory muscles are some of the important changes occurring due to non-enzymatic glocalization in DM [8]. The alveolar capillary network in the lung is a large micro-vascular unit and may be affected by microangiopathy due to hyperglycemia in DM which may impact cardiorespiratory fitness (CRF) and respiratory patterns in these patients [9]. Studies have reported lung spirometry parameters and diffusion capacity are decreased in patients with type 2 diabetes [10]. One of those, have reported significant deterioration of lung function and diffusing capacity in type 2 diabetes patients with poor glycemic control [11].

Stating further, CRF appraises an individual’s exercise capacity determined by maximum oxygen consumption (VO_2_ max measured in ml/kg/min) by the body which is directly linked to the integrated function of several body systems and may be a marker of total body health [12]. Low CRF is associated with an increased risk of cardiovascular disease among patients with type 2 diabetes [13]. Balducci and Cols et al. observed that increasing VO_2_ max by approximately 2 ml/kg/min can significantly reduce 10-year risk of coronary heart disease in these individuals [14]. Moreover, a 9% lower relative risk of all-cause mortality was shown among adult men with VO_2_ max of 1 ml/kg/min higher as compared to others [15]. Insulin sensitivity is closely associated with VO_2_ max and endothelial dysfunction bidirectionally [16, 17]. For example: individuals with better exercise capacity tend to exhibit improved insulin sensitivity and healthier endothelial function and vice versa [18]. Persons with type 2 diabetes mellitus (T2DM) have an impaired ability to carry out exercise even in the absence of clinically evident cardiovascular disease. A study reported peak oxygen uptake (VO_2_ peak) was reduced by approximately 20% in diabetic patients compared with non-diabetic controls matched for age and weight [19].

Impaired pulmonary functions and VO2 max levels have direct impact on physical fitness of individuals which may affect the general fitness including muscular strength, agility and flexibility [20] which again determine the exercising capacity of individuals [21]. Individuals with less VO_2_ max levels are prone to decrease muscular strength and muscle flexibility [22].

To what extend DM affects a person’s overall cardiorespiratory fitness, there is limited data available. The purpose of this research was to take an extensive view of pulmonary functions, cardiorespiratory fitness (VO_2_ max levels) and general fitness challenges faced by patients with type 2 DM in our population. In this study, we have also evaluated the association of pulmonary function, VO_2_ max levels and fitness level of DM participants with glycemic control and duration of onset of DM.

## Methods

### Study description

In this case-control study, a total of 200 participants were recruited from Dow University Hospital (DUH) Karachi Pakistan from January 2022 to July 2023.

### Ethical statement

The study was approved by Institutional Review Board (IRB) of DUH. The IRB approval reference number is IRB-1786/DUHS/Approval/2020. The participants were recruited in the study after written informed consent.

### Sample size calculation

Using NCSS PASS software, with 95% confident interval and 80% power of the test, the sample size came to be 100 for each group. The sampling technique was a purposive convenient type.

### Recruitment of participants

#### Diabetic (DM) group

One hundred diabetic patients were recruited from the department of Endocrinology of DUH. Participants of both genders having HbA1c >6.5% and age 18 and above were included in the study. Exclusion criteria were participants with any complication of DM, known history (based on verbal recollection) of acute viral infection or chronic (Hepatitis B / C / HIV) infection, history of active TB, asthma, and pregnancy.

#### Healthy control (HC) group

In total, 100 individuals with HbA1c <5.6 of either gender, age 18 or above were included in the study with the same exclusion criteria. HC group were either attendants of the patients or volunteers from the same hospital or Dow International Medical college.

#### Data collection

In all participants, at the time of recruitment, data regarding age, gender, BMI, HbA1c, family history of DM, and any history of comorbid conditions was recorded. Further data was collected as follows:

#### Spirometry and VO_2_ max levels

The spirometry was performed in willing participants with the help of a trained staff with portable MIR-Spirolab-III machine. Those who were able to complete the spirometry were further included in the study. Cut off value for obstructive respiratory pattern, FEV_1_/FVC <75–80% was considered. For restrictive respiratory patterns, FVC <80% was defined [23].

The VO_2_ max in ml/kg/min was calculated by heart rate ratio formula [24]. The formula 15.3× (maximum heart rate ÷ minimum heart rate) was applied. The heart rate ratio formula has been widely used in multiple studies. It has been validated for measurement of VO_2_ max in middle-aged and older adults [25] and also for the diabetic population [26].

#### Assessment of fitness

The fitness levels were measured by International Fitness scale (IFiS). IFiS represents a self-reported, straightforward fitness scale that has undergone validation in various studies [27]. Utilizing a 5-point Likert scale, the IFiS includes questions addressing five key fitness domains: overall fitness, cardiorespiratory fitness, muscular strength, agility, and flexibility. Alessandro Gatti et al. demonstrated that the IFiS score serves as a predictive indicator for objectively measured physical fitness [28].

### Statistical analysis

Statistical analysis was carried out using the Statistical Packages for Social Sciences (SPSS) version 22 and GraphPad PRISM version 8.1. The data was presented as median values. The Pearson’s Chi squared test was used to compare categorical data. While independent T test was used to compare the continuous data.

## Results

### a. Characteristics of participants

We included matched cases and controls in our study. There were 51% and 48% males from diabetes mellitus (DM) and healthy control (HC) groups respectively there was no gender difference in both groups (p = 0.118). There were 55% and 58% of participants with <50 years of age in DM and HC groups (p = 0.34). There was no BMI difference in either group (p = 0.189). The mean HbA1c of DM was 8.91 ± 1.69 and that of HC was 5.1 ± 0.3 (p<0.05; Table 1).

**Table 1:** Characteristics of study participants. N = 200 (DM = 100; HC = 100).

Variables		DM (n/%)	HC (n/%)	P value
Age (years)	≤50 years	55	58	0.34
	>50 years	45	46	
Gender	Male	51	48	0.118
	Female	49	52	
Family history of DM	Yes	23	18	0.81
	No	77	82	
BMI	Underweight	2	7	0.071
	Normal	14	39	
	Overweight	47	36	
	Obese	37	18	
Comorbidities	Yes	19	21	0.631
	No	81	79	
Years of diagnosis	1–6 years	55	NA	NA
	7 and above	45		
HbA1c (mean)		8.91 ± 1.69	5.5 ± 0	0.002**

The table shows the demographic characteristics and clinical history of study participants. The cases and controls were matched for age, gender, family history of DM, BMI, and comorbid conditions. Pearson’s Chi square was applied to check the difference in characteristics of groups. Independent T test was applied to for HbA1c difference in DM and HC. P value <0.05 was considered as significant at 95% CI. DM = diabetes mellitus; HC = healthy controls.

### b. Respiratory pattern among study participants

**Fig 1:**
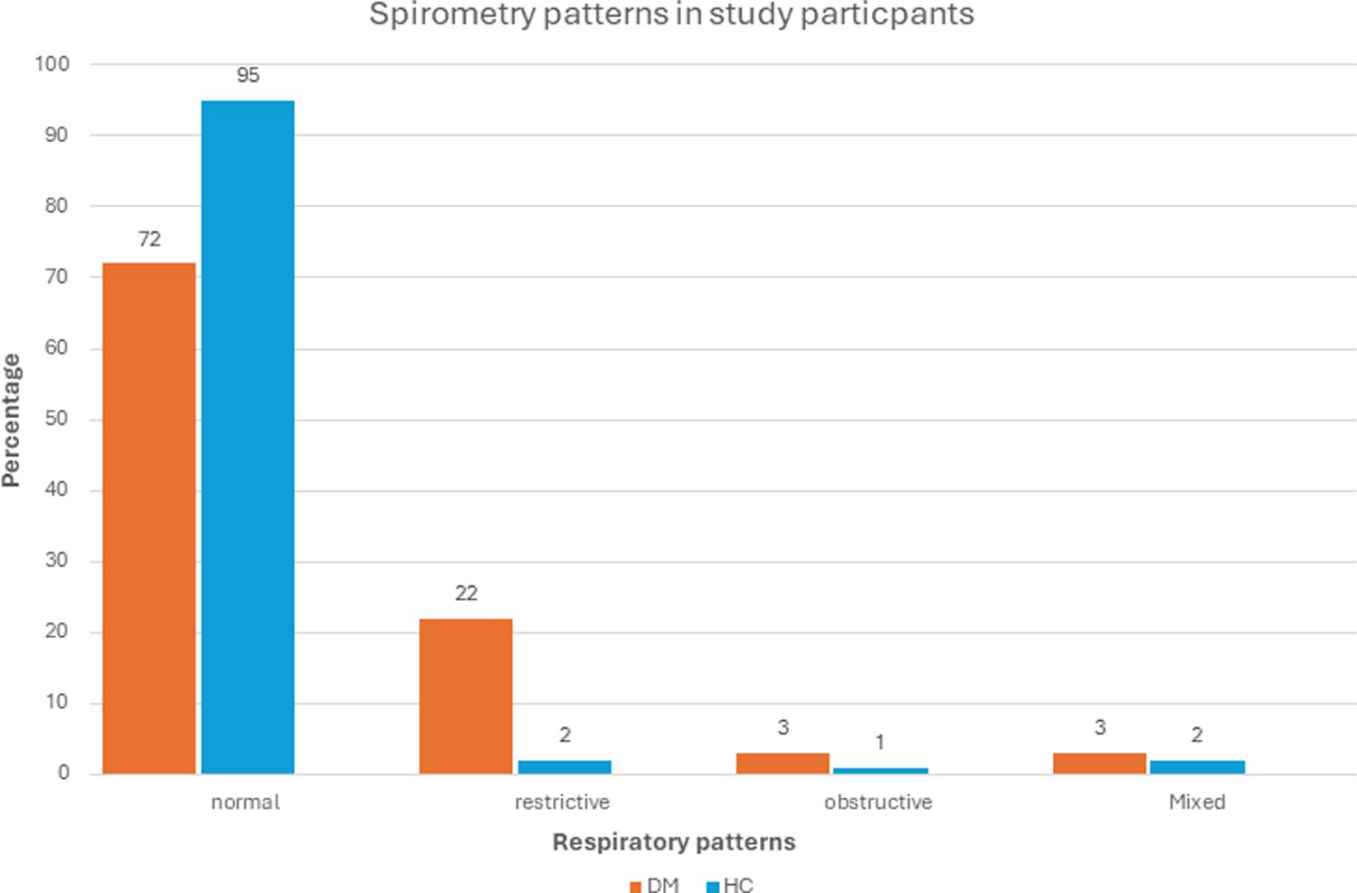
Respiratory patterns among study participants DM vs HC (n = 200; DM = 100, HC = 100). Cut off value for restrictive respiratory patterns, cut off FVC was <80% and for obstructive respiratory pattern, FEV_1_/FVC <75–80% was considered. Mixed was defined as low FVC and FEV_1_/FVC than predicted.

We found 22% and 2% of participants having restrictive respiratory pattern in DM and HC groups respectively (p<0.05). Normal respiratory pattern was found in 72% and 95% of DM and HC groups respectively (p<0.05). No difference was found with respect to obstructive or mixed respiratory patterns (p = NS; Fig 1).

### c. Association of restrictive respiratory pattern with glycemic control and years of DM onset

We compared the restrictive pattern of spirometry in DM with age, gender, BMI, comorbid conditions, years of onset of DM and glycemic control (HbA1c). Pearson’s Chi square test revealed a higher proportion of participants with restrictive respiratory pattern were having a higher BMI (p = 0.034), comorbid conditions (0.023), more years of onset of DM (0.001) and HbA1c higher than 8% (p = 0.002; Table 2).

**Table 2:** Association of spirometry patterns in DM with study variables. N = 100.

Variables	Subset	(n/%)	Spirometry patterns (n/%)				P value
			Normal n = 72	Restrictive n = 22	Obstructive n = 3	Mixed n = 3	
Age	≤50 years	55	42	10	1	2	0.711
	>50 years	45	30	12	2	1	
Gender	Male	51	37	11	1	2	0.567
	Female	49	35	11	2	1	
BMI	Underweight	1	1	1	-	-	0.034*
	Normal	14	10	2	1	1	
	Overweight	48	34	11	2	1	
	Obese	37	27	9	-	1	
Family history	Yes	23	8	12	1	2	0.682
	No	77	64	10	2	1	
Comorbid	Yes	19	5	14	-	-	0.023*
	NO	81	67	8	3	3	
Years on DM onset	1–6 years	55	45	6	2	2	0.001*
	7 and above	45	27	16	1	1	
HbA1C	≤8.0%	47	37	8	1	1	0.002*
	>8.0%	53	36	14	2	2	

Table shows association of respiratory patterns with BMI, comorbid conditions, years of onset of disease and HbA1c levels. Pearson Chi square was applied to look for the associations. P value <0.05 was considered as significant.

### d. Differential VO_2_ max among study participants

**Fig 2:**
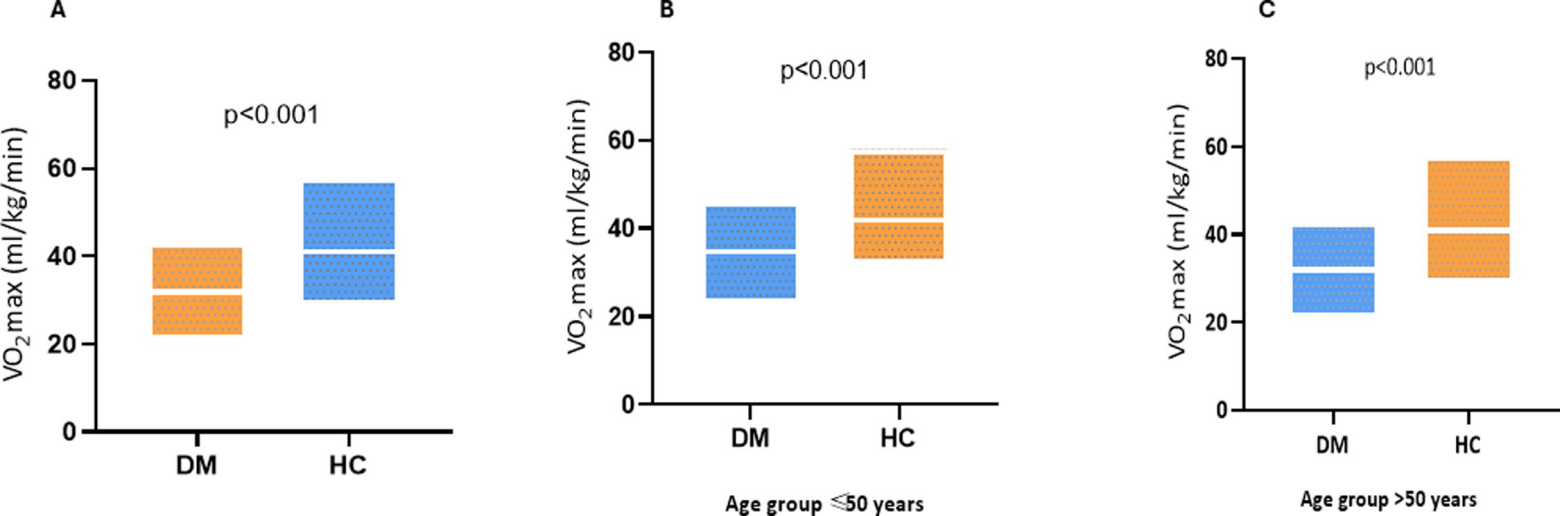
Comparison of VO_2_ max (ml/kg/min) between the DM and HC. (N = 200; DM = 100, HC = 100). A: Overall comparison of VO_2_ max. B: Comparison of VO_2_ max in age ≤50 years old in DM and HC. C: Comparison of VO2 max in age >50 years in DM and HC.

There was a significant difference in VO_2_ max levels between study groups. DM had lesser VO_2_ max (32.03 ± 5.3ml/kg/min) as compared to HC (41.91 ± 7.98ml/kg/min; p<0.001; Fig 2A). We also compared VO2 max levels in study groups with respect to age. There was decreased VO_2_ max levels in participants aged above 50 in both HC and DM groups and compared to ≤50 years participants (median VO2 max in DM ≤50 was 34ml/kg/min vs >50 years was 31ml/kg/min; Fig 2B and 2C).

### e. Association of VO2 max with glycemic control and years of DM onset

We further evaluated the association of VO_2_ max with study variables in DM group. We found females had lesser VO_2_ max as compared to males (p = 0.002). There was no significant association of VO_2_ max with BMI (p = 0.062). However, we found a significant association of VO_2_ max levels with glycemic control and year of onset of DM. Participants having diabetes with higher HbA1c and more years of disease onset were found to have lesser VO_2_ max as compared to those had less years of disease onset and HbA1c less than 8.0% (Table 3).

**Table 3:** Association of VO_2_ max with glycemic control and years of DM onset. N = 100.

Variables		(n/%)	VO_2_ max (mean)	SD	P value
Age	≤50 years	55	32.67	**6.42**	0.031*
	>50 years	45	29.39	**3.72**	
Gender	Male	51	31.25	**3.96**	0.002*
	Female	49	29.80	**3.617**	
BMI	Underweight	1	37.10	**-**	0.062
	Normal	14	31.50	**3.67**	
	Overweight	47	29.83	**3.185**	
	Obese	38	29.22	**3.86**	
Family history	Yes	23	29.25	**3.73**	0.29
	No	77	30.30	**3.95**	
Comorbid	Yes	19	28.31	**2.12**	0.038*
	NO	81	31.98	**3.19**	
Years on DM onset	1–6 years	55	32.77	**3.94**	0.0027*
	7 and above	45	28.25	**4.34**	
HbA1C	≤8%	47	32.78	**4316**	0.001*
	>8.1%	53	28.29	**4.97**	

Table shows association of VO_2_ max with Age, gender, comorbid conditions, years of onset of disease and HbA1c levels. Pearson Chi square was applied to look for the associations. P value <0.05 was considered as significant.

### f. Fitness of study groups on IFiS scale

#### i. Fitness of healthy controls

**Fig 3:**
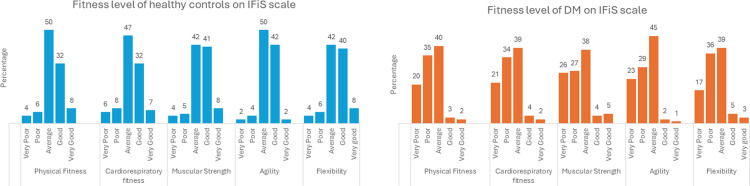
IFiS scale of fitness in study participants (N = 200). A: Figure shows fitness level of Healthy controls (n = 100) on a self-reported 5-point Likert Internation fitness scale (IFiS); B: IFiS Scale of Fitness in DM (n = 100). Fitness levels were reported on a self-reported 5-point Likert Internation fitness scale (IFiS).

On the 5-point Likert scale, 50% and 32% of HC reported to have average and good physical fitness respectively while 47% and 32% reported to have average and good cardiorespiratory fitness respectively. For muscular strength, agility, and flexibility, more than 40% of participants reported to have average and good on IFiS Likert scale (Fig 3A).

#### ii. Fitness of diabetic participants

On the same scale, 35% and 20% of participants reported to have poor and very poor physical fitness respectively while 34% and 21% of participants reported to have poor and very poor cardiorespiratory fitness respectively. Only 3 to 5% of participants were to have good or very good muscular strength, agility, and flexibility in this group (Fig 3B).

### g. Differential fitness of study groups

We then compared the differential fitness of DM and HC through Pearson’s Chi square test. We found a significant difference of physical fitness, cardiorespiratory fitness, muscular strength, agility, and flexibility on self-reported IFiS fitness scale (Table 4).

**Table 4:** Comparison of self-reported fitness on IFiS scale components between DM and HC (n = 200; DM = 100, HC = 100).

		Diabetes mellitus n = 100	Healthy controls N = 100	P value
Variables of IFiS scale	Scale	n/%	n/%	
Physical Fitness	Very Poor	0	2	<0.05
	Poor	4	6	
	Average	91	52	
	Good	5	32	
	Very Good	0	8	
Cardiorespiratory fitness	Very Poor	0	6	<0.05
	Poor	38	12	
	Average	60	47	
	Good	2	32	
	Very Good	0	3	
Muscular Strength	Very Poor	0	0	<0.05
	Poor	27	10	
	Average	69	42	
	Good	4	46	
	Very Good	0	2	
Agility	Very Poor	0	0	<0.05
	Poor	20	4	
	Average	78	50	
	Good	2	44	
	Very Good	0	2	
Flexibility	Very Poor	0	0	<0.05
	Poor	38	10	
	Average	57	42	
	Good	5	40	
	Very good	0	8	

Table shows differential fitness level of HC and DM with Pearson Chi square test. P value <0.05 was considered as significant.

## Discussion

Our research has shown a substantial decrease in respiratory pattern, VO_2_ max levels and physical fitness in diabetic patients as compared to healthy controls which are also associated with glycemic control and duration of disease.

We found 22% of DM with restrictive respiratory patterns which was found to be associated with BMI. Earlier research has shown that a higher proportion of participants with restrictive spirometry patterns (RSP) had a raised body mass index [29]. In addition, we found the impact of duration of disease on restrictive respiratory patterns which was also found in a study from Korea which highlighted the progressive deterioration of lung function in diabetics over time [30]. An other study revealed that compared to people without diabetes, people with diabetes had significantly decreased lung functions [31]. Another important finding in our study was that patients with a HbA1c level above 8% (uncontrolled hyperglycemia) were more prone to have impaired breathing patterns. This result is consistent with Maan, Meo [32] study which found that patients with type 2 diabetes were more likely to develop restrictive breathing patterns and impaired lung function and this effect was found more in participants with uncontrolled hyperglycemia. However, according to a study by Gläser, Krüger [33], RSP was present in one-third of those with type 2 diabetes which is higher than our reported percentage (22%). Longer duration of DM and uncontrolled hyperglycemia leads to reduced elastic recoil, reduced lung volume, diminished respiratory muscle performance, chronic low-grade inflammation, decrease in pulmonary diffusion capacity for carbon monoxide, which are some of the important changes occurring due to non-enzymatic glocalization. The alveolar capillary network in the lung is a large micro-vascular unit and may be affected by microangiopathy due to hyperglycemia in DM which may impact cardiorespiratory fitness (CRF) and respiratory patterns in these patients.

Failing to sustain sufficient levels of physical activity and CRF increases the risk of cardiovascular complications. Previous studies have highlighted that elevated regular physical activity and cardiorespiratory fitness (VO_2_ max) levels are associated with a diminished risk of coronary heart disease [34, 35]. Wei et al. specifically reported a noteworthy association between low cardiorespiratory fitness and impaired fasting glucose, T2DM, emphasizing its role as an independent predictor of all-cause mortality in men with T2DM [36, 37]. Our research found a statistically significant difference in maximal oxygen consumption (VO_2_ max) between healthy controls (HC) and patients with type 2 diabetes mellitus (DM) which was also reported by Awotidebe, Adedoyin [38]. Caron, duManoir [39] also reported that type 2 diabetics generally had a lower maximal oxygen consumption than non-diabetics. Also, a significant association of VO_2_ max levels with glycemic control and year of onset of DM is in line with the past literature [40]. Many diabetes-related complications and duration lead to decreased cardiorespiratory fitness, which in turn leads to declined maximal oxygen saturation, including reduced exercise capacity, peripheral vascular dysfunction, and poor oxygen utilization at the tissue level. In addition, the present study found lower maximal oxygen saturation in the diabetic group and in the 50+ age group, which is in accordance with previous studies showing that aerobic capacity decreases with age in different population groups [41]. Ozaki, Loenneke [42] also mentioned that the VO_2_ max decreased with time; after the age of 30, it dropped about 2% per year. This pattern, which showed a decline in aerobic capacity, was present in several demographics, including diabetics. Another study discussed that a person’s aerobic capacity naturally decreased with age due to physiological changes such as atrophying muscles, reduced cardiac output, and worse lung function [43]. The current research supports the notion that age affects cardiorespiratory outcomes in type 2 diabetics, as shown by the comparison of maximal oxygen saturation between individuals in the diabetes group who are younger (≤50 years) and older (>50 years).

Our study showed that women had lower VO_2_ max values than men, which is consistent with previous findings [44]. Past literature revealed that, on average, men had higher maximal oxygen saturation than women. This gender difference may be attributed to a variety of factors, including muscle mass, hemoglobin levels, and the effects of hormones on cardiovascular function [45]. In the present study, no significant association was found between maximal oxygen saturation and body mass index (BMI), which is similar to the results of prior research, which showed no correlation association [40]. However, Li, Yang [46] noted the negative association between maximal oxygen saturation and body mass index that can be influenced by many factors, including age, fitness level, and body fat distribution. Furthermore, the results of a significant association between maximal oxygen saturation and glycemic control (as measured by HbA1c) in the current study has been supported by studies. A research found a significant relationship between maximal oxygen saturation and glycemic control [40]. This correlation as revealed by Azhar, Khan [47] was due to the negative effects of chronic hyperglycemia on cardiovascular function and performance. Also, the lower maximal oxygen saturation (VO_2_ max) values in participants with longer duration of diabetes are consistent with results reported in the literature [48].

In our study, we used the IFiS scale to assess various components of fitness, revealing significant disparities in fitness components between the DM and HC groups. The HC exhibited notably higher fitness components as compared to diabetic group, emphasizing the potential impact of diabetes on fitness components. Data was further explored to find the association between physical fitness, muscular strength, agitation, flexibility, and their effect on cardiorespiratory fitness. In our study within the diabetic group, individuals with lower cardiorespiratory fitness generally exhibited lower levels of physical fitness, muscular strength, and flexibility compared to the healthy group. A cohort study indicated that individuals engaging in flexibility and muscle-strengthening activities showed increased CRF, lower BMI, higher aerobic activity participation, lower total cholesterol, and a lower prevalence of diabetes and hypercholesterolemia [49]. A review reported that in various population studies, there exists a moderate correlation between muscular fitness and CRF [50, 51]. They reported that the literature suggests that maintaining sufficient muscular strength, muscular endurance, and flexibility can enhance the ability to perform daily activities and engage in physical exercise, potentially contributing to the maintenance of CRF [52]. CRF and glycemic control are linked with each other bidirectionally. Better CRF can provide enough strength to carry out physical activities which can control hyperglycemia and provide better respiratory fitness. However, controlled glucose levels will least affect CRF and can provide enough strength to muscles to perform physical activity.

## Conclusion

Our study reports restrictive respiratory pattern, decreased VO_2_ max levels and physical fitness level in considerable number of individuals with DM which is associated with glycemic control and duration of disease. Hence consideration of pulmonary function test should be encouraged along with glycemic control during the management of patients with diabetes.
